# Using a Standard Infrarenal Bifurcated Device as a Quadruple-Fenestrated Physician-Modified Endograft for Complex Abdominal Aortic Aneurysms—A Simulation Study

**DOI:** 10.3390/jcm14124249

**Published:** 2025-06-15

**Authors:** Artúr Hüttl, András Szentiványi, Ákos Bérczi, Bendegúz Juhos, Fanni Éva Szablics, Péter Osztrogonácz, Judit Csőre, Sarolta Borzsák, Csaba Csobay-Novák

**Affiliations:** 1 Semmelweis Aortic Center, Department of Interventional Radiology, Heart and Vascular Center, Semmelweis University, Városmajor u. 68., H-1122 Budapest, Hungary; huttl.artur@semmelweis.hu (A.H.);; 2 Semmelweis Aortic Center, Department of Vascular and Endovascular Surgery, Heart and Vascular Center, Semmelweis University, Városmajor u. 68., H-1122 Budapest, Hungary

**Keywords:** physician-modified endograft, endovascular, aortic, aneurysm

## Abstract

**Background/Objectives**: We sought to demonstrate the versatility and economy of physician-modified endograft (PMEG) fenestrated endovascular aortic repair (FEVAR) based on the Treo (Terumo Aortic) platform for patients referred for custom-made device (CMD) FEVAR due to a complex abdominal aortic aneurysm (CAAA). Endovascular planning was performed utilizing a standardized design incorporating all visceral arteries with a low supra-celiac landing zone. The pure cost of the aortic components was compared between the PMEG and CMD designs. **Methods**: A total of 39 consecutive patients treated with CMD FEVAR due to a CAAA between September 2018 and December 2023 were recruited at a tertiary vascular center for a retrospective evaluation. Endovascular planning was performed on readily available computed tomography angiography (CTA) datasets using 3Mensio Vascular (Pie Medical Imaging) software. The actual cost of the major components was compared between the implanted CMD platform produced by Cook and the planned Treo-based PMEG repair. **Results**: A total of 155 fenestrations were planned on 3 triple-, 34 quadruple-, and two quintuple-fenestrated devices. The 90 mm distance between the proximal edge and the flow divider of the 120 mm long main body of the Treo graft allowed for the placement of all necessary fenestrations of the target arteries without the need to reduce the 3 cm supra-celiac landing zone while also preserving a safety distance of >1 cm to the flow divider. The costs of the components were EUR 33896 for CMD and EUR 8878 for a PMEG. **Conclusions**: This retrospective study suggests that a quadruple-fenestrated PMEG based on the Treo bifurcation is a highly versatile alternative with a significant price advantage over custom-made devices for the treatment of complex abdominal aortic aneurysms.

## 1. Introduction

Over the past decade, fenestrated endovascular aortic repair (FEVAR) with patient-specific custom-made devices (CMDs) has become widely accepted as a first-line treatment for complex abdominal aortic aneurysms (CAAAs) [1]. The physician-modified endograft (PMEG) technique was created to overcome the limitations of CMDs, such as their limited availability, associated with either country- or center-specific access restrictions or their lengthy lead times, the latter being an obstacle especially in urgent settings, often taking up to 12 weeks from therapeutic decision to the actual implantation [2].

Several devices are currently being used as a platform for PMEG FEVAR, such as Valiant Captiva or Endurant stent graft systems (Medtronic, Dublin, Ireland) or the Treo bifurcation device (Terumo Aortic, Inchinnan, UK), mostly based on the preference of the primary operator [3,4,5]. Each device has its drawbacks and advantages regarding delivery system (re)usability, entry profile, strutless area, main-body length, ease of reloading, and other issues. The Treo bifurcation device offers several benefits for such uses, including a 9 cm long main trunk; large strut-to-strut distances, resulting in large strutless areas, facilitating fenestration positioning without the need to modify the struts; and a low entry profile [6]. As it is a bifurcation device, there is no need to extend it with an aortic component, which might add to the overall risk of repair [6,7]. Several centers have reported their initial experiences with applying this device to a large number of patients; however, the versatility of this approach is yet to be defined [8,9,10]. Therefore, this study aimed to demonstrate the versatility of PMEG FEVAR based on the Treo platform in patients referred for CMD FEVAR due to a CAAA. Endovascular planning was performed utilizing a standardized design incorporating all visceral arteries with a low supraceliac landing zone. The pure cost of the aortic components was compared between the PMEG and CMD designs.

## 2. Materials and Methods

Consecutive patients treated with CMD-FEVAR to address a CAAA between September 2018 and December 2023 were recruited at a tertiary vascular center for a retrospective evaluation. This study was conducted in accordance with the Declaration of Helsinki, and the protocol was approved by the local ethical committee (SE RKEB 129/2024).

Endovascular planning was performed on readily available computed tomography angiography (CTA) datasets using 3Mensio Vascular (Pie Medical Imaging BV, Maastricht, The Netherlands) software. After automatic vascular segmentation, the centerline was edited in order to minimize imprecision, which is often associated with elongations. The proximal edge of the device was used as a reference point, aiming for a 30 mm landing zone above the celiac axis (the low supra-celiac landing in zone 5). The exact position of the proximal edge was often modified to preserve as many intercostal arteries as possible to minimize the risk of spinal cord ischemia. Distances from the proximal edge to the fenestrations, from the proximal edge to the flow divider, and from the flow divider to the iliac bifurcation were measured. The 120 mm long Treo main body (Catalog Number: 28-B2-xx-120S) was used, which measures 90 mm from the proximal edge to the flow divider. Its diameter was set according to the supraceliac aortic diameter utilizing a 10 to 15% oversize [11]. Fenestrations and iliac limbs were planned in a standard fashion identical to that for CMDs. Parameters of the necessary limbs were collected for both the CMD and the PMEG designs. Limbs for the PMEG repair were planned with Treo limbs by default. If the required limb was longer than the longest available Treo limb, 199 mm long Endurant (Medtronic, Minneapolis, MN, USA) limbs were also considered as a replacement, as the Treo and the Endurant platforms share a similar design in regard to the contralateral gate and the design of the proximal overlap zone of the limbs, making them technically interchangeable. The actual costs of the devices for the CMD repairs were determined, and the costs of the devices needed for each PMEG repair were calculated. Bridging stent cost was not added to the overall cost, as the sums were the same regardless of the type of repair. Cannulation of the target artery and the deployment of a flared bridging stent was considered challenging if the lowest fenestration was within proximity (20 mm) of the flow divider of the main body.

Continuous parameters are expressed as means ± standard deviations (SDs), while categorical variables are delineated as counts and percentages. Statistical analyses and graphical representations were conducted using StataCorp LLC Stata (College Station, TX, USA, version 18).

## 3. Results

A total of 39 patients (30 males, 76.5 ± 6.8 years) with CAAAs were evaluated for FEVAR with a CMD and a PMEG (Table 1).

**Figure 1:**
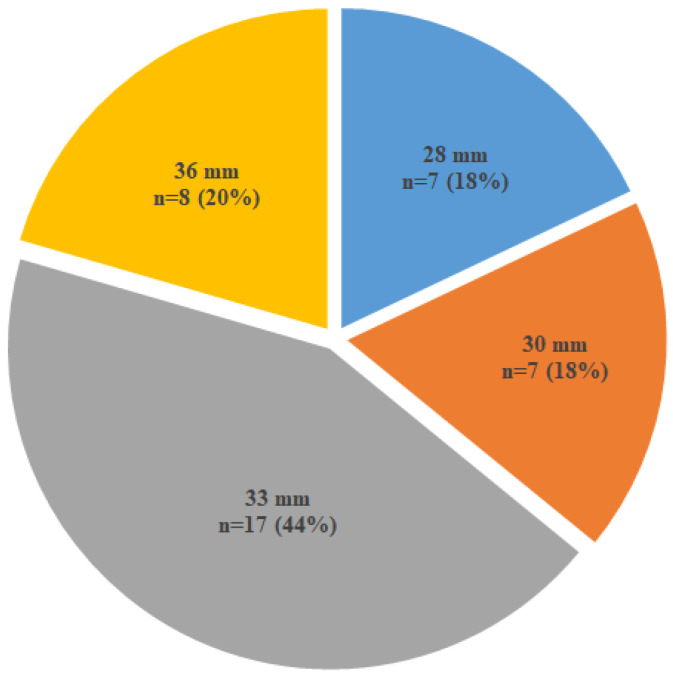
Distribution of the main bodies implanted.

A total of 7 (17.9%) of the 28 mm, 7 (17.9%) of the 30 mm, 17 of the 33 mm (43.6%), and 8 of the 36 mm main bodies (20.5%) were implanted (Figure 1).

A total of 155 fenestrations were planned on 3 triple-, 34 quadruple-, and 2 quintuple-fenestrated devices (Table 2). The 90 mm distance between the proximal edge and the flow divider of the 120 mm long main body of the Treo graft allowed for the placement of all necessary fenestrations of the target arteries without the need to significantly reduce the 3 cm supra-celiac landing zone.

**Figure 2:**
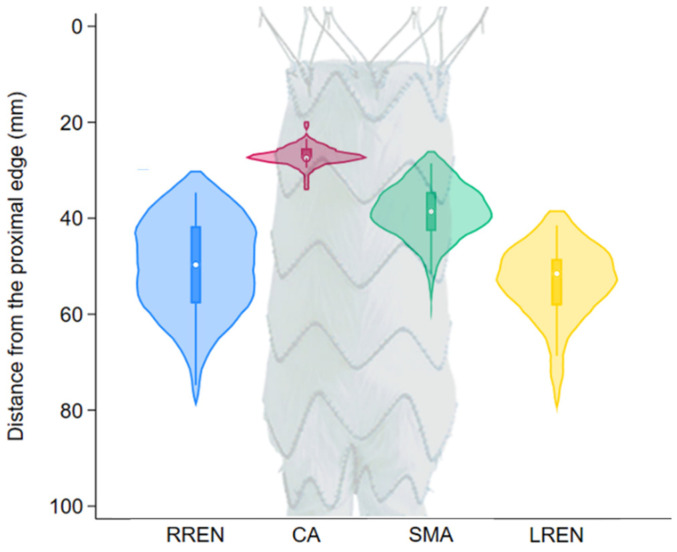
Violin plot of the positions of the fenestrations on a 120 mm Treo main body. RREN: right renal artery (blue); CA: celiac axis (red); SMA: superior mesenteric artery (green); LREN: left renal artery (yellow).

Figure 2 shows the distribution of the positions of the fenestrations on a 120 mm main body. The distance from the proximal edge was 27.6 ± 2.1 mm for the celiac axis, 39.4 ± 6.1 mm for the superior mesenteric artery, 51.1 ± 10.4 mm for the right renal artery, and 54.4 ± 8.3 mm for the left renal artery. In four (10.2%) cases, one or both of the renal fenestrations were located less than 2 cm above the flow divider, a situation that might have led to a challenging cannulation and/or flaring as per our definition.

Thirty-eight (49%) limb extensions required a single Treo limb as a distal extension to achieve a safe landing zone in the common iliac artery. Forty extensions (51%) required an additional limb extension, as the necessary device was longer than the longest available Treo limb (160 mm). All of these cases required a device that was less than 200 mm, a length theoretically feasible for the implantation of the longest Medtronic Endurant limb extension (199 mm). Regarding limb diameters, 9 mm limbs were not planned, whereas an 11 mm limb was required in 3 cases (4%), a 13 mm limb was required in 27 cases (35%), a 15 mm limb was required in 19 cases (24%), a 17 mm limb was required in 14 cases (18%), a 20 mm limb was required in 8 cases (10%), and a 24 mm limb was required in 7 (9%) instances.

The cost of the aortic components was EUR 33,896 for CMD using the Cook platform, whereas it was EUR 12,492 ± 2464 for PMEG using the Treo platform. If Medtronic Endurant limbs were used as a replacement instead of Treo and needed to be extended, the overall component price of the PMEG repair could have been further reduced to a fixed price of EUR 8878, given that a main body and two limbs were used constantly.

## 4. Discussion

Current guidelines do not recommend the PMEG technique unless it is used to address an urgent situation or as part of a study [1]. There is a growing body of evidence in support of this technology, making a future change in the respective recommendations very likely. In this study, we aimed to evaluate the feasibility of a PMEG FEVAR based on the Treo bifurcation in regard to fenestration positioning. We found that the long main body of this device allowed for the comfortable positioning of the 155 target arteries of the 39 patients evaluated in the mid-portion of the trunk while maintaining a low supra-celiac landing zone of 3 cm. A significant reduction in device-related costs could be achieved.

A common argument against the use of the PMEG technique is the non-standardized nature of such procedures. Indeed, the techniques of the leading centers are rather different regarding both the general approach and the technical details of the procedures [4,10,12]. However, the fact that the different devices and techniques are currently used for PMEG repair is not purely a drawback; it can also be seen as an advantage: contrary to CMDs, where we have to use the same platform regardless of the situation, the PMEG technique offers great flexibility and versatility with the potential for rapid development and changes in design to better suit our needs. Non-bifurcated aortic devices offer great flexibility for positioning the fenestrations while also being relatively easy to reload. Increasing reports have resulted in rather sophisticated workflows following this approach, which is still the preferred technique for several centers worldwide [5]. Bifurcated devices, however, have advantages over straight components. Having been used already during the infancy of the PMEG technique, standard EVAR devices can successfully overcome some of the limitations of straight aortic endografts [2]. Abdominal devices have a substantially lower profile (18–19 F vs. 22–24 F) that helps to keep repairs minimally invasive. Moreover, as they are inherently bifurcated, there is typically no need to extend them with a second aortic component, unlike the approach often required in CMD FEVAR. While bifurcated CMDs are available, they are less commonly utilized in practice. This could be an issue during repair, as it was shown that the nosecone of the bifurcated extension of a CMD might interfere with the flared bridging stents, possibly necessitating reintervention. In a recent study by Karelis et al., it was demonstrated that a no-cross concept utilizing a short tip distal extension component during CMD FEVAR was associated with a lower rate of intraoperative fenestration-related adverse events [7]. However, this elegant solution solves a problem that is virtually nonexistent if the fenestrated device is a bifurcated component. An extra overlap zone that is unnecessary might also compromise the durability of repair in the long run, given the inherent—although rather low—risk of component separation and a consequential type III endoleak.

The TreoFit system (Terumo Aortic, Inchinnan, UK), which was recently introduced, is intended to facilitate the PMEG workflow. The kit simplifies procedural workflows and is a step forward regarding the standardization of this technique. Nevertheless, there is currently no published literature available regarding its clinical application.

Current abdominal endograft designs differ in terms of the length of the main body; the two most commonly used devices share a short and fixed-length main body (4 cm for the Excluder device [Gore Medical, Flagstaff, AZ, USA] and 54 mm for the Endurant device [Medtronic]). This makes the use of these devices for quadruple-fenestrated repairs rather challenging [3,13]. Cook Medical and Terumo Aortic followed a different approach regarding main-body design, allowing the operator to choose from a wide range of main body lengths. Although the decision to follow this design was most likely unrelated to PMEG movement, the versatility of this long main body is very useful for these purposes, as discovered during the early years of the PMEG technique [2]. Although there are techniques for handling a short main body with four fenestrations, the lowest renal fenestration will often be close to the proximal edge, a situation that might be challenging to manage during a procedure. Also, the supraceliac landing zone will be minimal, making it rather difficult to use as a landing zone for a future TEVAR in case of a late type 1 endoleak. In our approach, consisting of using a 3 cm supraceliac landing zone, we followed an anticipative strategy to make a potential late reintervention easier. The lowest renal fenestration was within 2 cm of the flow divider in 10% of our cases; however, none of these were within 1 cm of the flow divider, making a challenging situation or an adverse event during the repair very unlikely.

For about 50% of our cases (19 out of 39 cases), the longest available limb on the Treo platform was too short. This issue was caused by the supra-celiac position of the main body. A trivial solution to this is the use of a distal extension with a second limb; however, one could also implant an Endurant limb instead (Medtronic), which offers a 199 mm limb length that would have been enough for all these cases. Being an Instructions For Use (IFU) application per se, the Treo and Endurant limbs are practically interchangeable: the devices share design similarities regarding the contralateral gate, having the same diameter (14 mm) and length (30 mm). The structures of the limbs are also very similar between the two platforms, making the lock–stent mechanism of the Treo gate act the same regardless of whether it is part of a Treo or Medtronic limb. The only difference is the proximal diameter of the limb: 15 mm for the Treo (7.1% oversize in a 14 mm gate) and 16 mm for the Endurant (14.3% oversize in a 14 mm gate)—a difference that is very unlikely to cause any issues. We believe the additional risk of using this off-label limb is very similar to that posed by a second limb extension, posing the inherent risk of a late disconnection at the extra overlap zone—a very low risk. However, the extra cost of the additional limb is non-negligible, making the use of the Endurant limb extension in such occasions rather appealing.

Our analyses might also help to build up stock that is ideal for urgent scenarios. We found that the small main bodies (20 mm, 22 mm, 24 mm, and 26 mm) were not needed in our cases, making the trio of the 30 mm, 33 mm, and 36 mm diameter, 120 mm long main bodies an ideal stock for almost every situation. Short limbs (80 mm, 100 mm, and 120 mm) were not used either, making a wide range of diameters available in 140 and 160 mm lengths ideal.

The cost of the devices showed a huge difference in favor of PMEGs, something that was expected and cannot be overlooked. This difference further increased if Endurant limbs were preferred instead of an additional Treo limb. However, the pricing of such standard and custom-made devices differs from country to country and center to center, making our findings less easily generalizable.

An important issue remains to be addressed before we can conclusively assert that the Terumo Treo graft is universally compatible with individual patient anatomy: the arrangement of the stent struts. An investigation into this matter would require access to the engineering schematics of the different Treo main bodies, which we do not have. Thus, this research does not yield a response to this question. However, based on our clinical experience, the incidence of such interference with the struts is very low with respect to the Terumo Treo main body and can usually be managed with very little offset of the affected fenestration, leading to a minimal shuttering effect that is clinically irrelevant during the implantation. Nevertheless, future experiments might validate this clinical observation.

There are various limitations to this study. First, its retrospective and single-center nature allows for selection bias and restricts generalizability. Second, our report focuses on feasibility and cost comparison without reporting clinical outcomes such as procedural technical success, complications, or the mid- to long-term durability of the repair, as it is a simulation study and these outcomes are already reported in the literature. Third, it lacks a formal cost-effectiveness analysis, so overall economic value could not be evaluated when considering clinical outcomes and quality-adjusted effects. Finally, the prices of the devices and their availability vary across regions and institutions, thereby limiting the generalizability of our cost comparisons.

## 5. Conclusions

This retrospective simulation-based feasibility study demonstrates that a quadruple-fenestrated physician-modified endograft based on the Treo bifurcation could be a highly versatile alternative with a possibly significant cost benefit for custom-made devices used for the treatment of complex abdominal aortic aneurysms. The device’s design allowed for the placement of the fenestrations of all the target arteries in the mid-part of the main body without the need to compromise the supra-celiac landing zone. Thus, a number of components’ overlap zones could be reduced, which might offer a durability benefit in the long run.

## Figures and Tables

**Table 1:** Patient demographics.

Number of patients	39
Male sex	30 (76.9%)
Age (year)	76.5 ± 6.8
Aneurysm diameter (mm)	67.2 ± 13.1

**Table 2:** Distribution of triple-, quadruple-, and quintuple-fenestrated devices.

Fenestrations per Device	Number of Devices	Total Number of Fenestrations
3	3	9
4	34	136
5	2	10
Total	39	155

## Data Availability

The original contributions presented in this study are included in the article. Further inquiries can be directed to the corresponding author(s).

## Abbreviations

The following abbreviations are used in this manuscript:

FEVAR	Fenestrated endovascular aortic repair
CMDs	Custom-made devices
CAAAs	Complex abdominal aortic aneurysms
PMEG	Physician-modified endograft
RREN	Right renal artery
CA	Celiac axis
SMA	Superior mesenteric artery
LREN	Left renal artery
